# Tonic Control of Secretory Acid Sphingomyelinase Over Ventral Hippocampal Synaptic Transmission and Neuron Excitability

**DOI:** 10.3389/fncel.2021.660561

**Published:** 2021-04-09

**Authors:** Chih-Hung Lin, Johannes Kornhuber, Fang Zheng, Christian Alzheimer

**Affiliations:** ^1^Institute of Physiology and Pathophysiology, Friedrich-Alexander-University Erlangen-Nürnberg, Erlangen, Germany; ^2^Department of Psychiatry, University Hospital Erlangen, Friedrich-Alexander-University Erlangen-Nürnberg, Erlangen, Germany

**Keywords:** acid sphingomyelinase, ventral hippocampus, pyramidal cells, action potentials, EPSC, IPSC

## Abstract

The acid sphingomyelinase (ASM) converts sphingomyelin into ceramide. Recent work has advanced the ASM/ceramide system as a major player in the pathogenesis of major depressive disorder (MDD). Indeed, ASM activity is enhanced in MDD patients and antidepressant drugs like fluoxetine act as functional inhibitors of ASM. Here, we employed the specific ASM inhibitor ARC39 to explore the acute effects of the enzyme on hippocampal synaptic transmission and cell excitability in adult mouse brain slice preparations. In both field potential and whole-cell recordings, ARC39 (1–3 μM) enhanced excitatory synaptic input onto ventral hippocampal CA1 pyramidal cells. The specificity of drug action was demonstrated by its lacking effect in slices from ASM knockout mice. In control condition, ARC39 strongly reduced firing in most CA1 pyramidal cells, together with membrane hyperpolarization. Such pronounced inhibitory action of ARC39 on soma excitability was largely reversed when GABA_A_ receptors were blocked. The idea that ARC39 recruits GABAergic inhibition to dampen cell excitability was further reinforced by the drug’s ability to enhance the inhibitory synaptic drive onto pyramidal cells. In pyramidal cells that were pharmacologically isolated from synaptic input, the overall effect of ARC39 on cell firing was inhibitory, but some neurons displayed a biphasic response with a transient increase in firing, suggesting that ARC39 might alter intrinsic firing properties in a cell-specific fashion. Because ARC39 is charged at physiological pH and exerted all its effects within minutes of application, we propose that the neurophysiological actions reported here are due to the inhibition of secretory rather than lysosomal ASM. In summary, the ASM inhibitor ARC39 reveals a tonic control of the enzyme over ventral hippocampal excitability, which involves the intrinsic excitability of CA1 pyramidal cells as well as their excitatory and inhibitory synaptic inputs.

## Introduction

Acid sphingomyelinase (ASM) is a phosphodiesterase, which hydrolyzes the membrane constituent sphingomyelin to ceramide and phosphorylcholine. Ceramide serves as a major hub in sphingolipid metabolism and signaling, and has been recognized as a major player in infection and inflammation, and, more recently, in depression (Kornhuber et al., 2005, 2014; Gulbins et al., 2013, 2015; Jernigan et al., 2015; Dinoff et al., 2017). Although encoded by a single gene in mammals (*SMPD1*), ASM comes in two distinct forms, which can be differentiated in several respects. Lysosomal ASM (L-ASM) is localized primarily inside lysosomes, where pH is close to the enzyme’s optimum (4.5–5.0). The other form, called secretory ASM (S-ASM), is endowed with a complex *N*-glycosylation pattern, which results in the sorting of the enzyme in a constitutive secretory pathway leading to its release into the extracellular space (Kornhuber et al., 2015). Unlike L-ASM, which has a half-life of 4–5 h, S-ASM is unusually stable and retains its full enzymatic activity over at least 25 h (Kornhuber et al., 2015). It is worth noting that, particularly under conditions of cellular stress and concomitant elevations in intracellular Ca^2+^, lysosomes might fuse with the cell membrane and release ASM, representing a second pathway through which the enzyme might reach the extracellular space (Kornhuber et al., 2015).

Overactivity of the ASM/ceramide system has been implicated in the pathogenesis of major depressive disorder (MDD) (Kornhuber et al., 2005; Gulbins et al., 2013). ASM-overexpressing (oeASM) mice exhibit enhanced anxiety- and depression-like behavior (Gulbins et al., 2013). The phenotype has been linked to pathological rises in ceramide levels, which have a number of detrimental effects on hippocampal structure and function, including atrophy, impaired control of the HPA axis, and suppression of adult neurogenesis in the dentate gyrus (Gulbins et al., 2013). Notably, the therapeutic potential of widely used antidepressant drugs such as amitriptyline and fluoxetine has been attributed to their ability to act as functional inhibitors of ASM (FIASMAs) (Kornhuber et al., 2009, 2010). These amphiphilic drugs accumulate in lysosomes and detach L-ASM from the inner lysosomal membrane, leading to subsequent proteolytic degradation of the enzyme, which in turn normalizes the pathologically enhanced ceramide levels (Kornhuber et al., 2009). According to this scheme, FIASMAs should primarily target L-ASM, without appreciable inhibition of S-ASM. The apparent preponderance of L-ASM over S-ASM with respect to CNS disorders received also support from a transgenic mouse line, which retained residual L-ASM activity (11–18% of wt activity) in the brain, but completely lacked S-ASM activity (Marathe et al., 2000). These mice showed no signs of severe neurological dysfunction and brain pathology (Marathe et al., 2000). It should be kept in mind, however, that oeASM mice have a 10-fold higher activity of S-ASM in the CSF compared to wt controls (Mühle et al., 2013), suggesting that overactivity of the secretory form of the enzyme might also contribute to MDD pathogenesis. Thus, at the moment the relative impact of the two forms of ASM on the workings of brain circuits involved in MDD remains largely elusive.

To address this issue, we interrogated the function of ASM in ventral hippocampus, with a particular focus on the secretory form. Several lines of anatomical, electrophysiological and behavioral evidence argue for a functional segregation of the hippocampus along its longitudinal axis. Whereas the dorsal part preferentially processes spatial information and serves other cognitive functions, the ventral part is preferentially involved in the regulation of emotionality and affective behavior, and has therefore garnered particular attention in studies on anxiety and depression (Bannerman et al., 2004; Fanselow and Dong, 2010; Strange et al., 2014). Using the highly selective ASM blocker ARC39 (Roth et al., 2009; Arenz, 2010; Naser et al., 2020) which, due to its strong negative charge at physiological pH should not appreciably permeate the cell membrane during brief applications of ≤10 min, we made the unexpected finding that the secretory form of the enzyme exerts a tonic regulatory effect on synaptic transmission and pyramidal cell firing in area CA1 of mouse hippocampus.

## Materials and Methods

Male and female adult (2–4 months old) wild-type mice (wt) with C57BL/6J background and ASM knockout (ASM-KO) mice (Horinouchi et al., 1995; Gulbins et al., 2013) were used for experiments. Animals were housed under standard conditions. All experiments were conducted in accordance with the Animal Protection Law of Germany and the European Communities Council Directive of November (1986/86/609/EEC) and were approved by the local government of Lower Franconia.

Horizontal ventral hippocampal slices (350 μm thick) were prepared from mice anesthetized with sevoflurane. Brain slices were cut in ice-cold sucrose-based artificial cerebrospinal fluid (aCSF) containing (in mM) 75 sucrose, 87 NaCl, 3 KCl, 0.5 CaCl_2_, 7 MgCl_2_, 1.25 NaH_2_PO_4_, 25 NaHCO_3_, and 10 D-glucose. Slices were incubated in the same solution for 10 min at 35°C, and then maintained in aCSF containing (in mM) 125 NaCl, 3 KCl, 1 CaCl_2_, 3 MgCl_2_, 1.25 NaH_2_PO_4_, 25 NaHCO_3_ and 10 D-glucose at room temperature for at least 2 h before being used. Individual slices were transferred to a submerged chamber that was mounted on the stage of an upright microscope (Olympus BX50WI), and perfused with normal aCSF with 1.5 mM MgCl_2_ and 2.5 mM CaCl_2_ at 30–32°C, unless otherwise stated. All solutions were constantly gassed with 95% O_2_–5% CO_2_. Contrast-enhanced IR camera was used to visualize CA1 pyramidal cells from ventral hippocampus (VH).

Extracellular field potentials were recorded in stratum radiatum of hippocampal CA1 region using pipettes filled with modified aCSF (to avoid pH change). In the presence of GABA_A_ receptor antagonist picrotoxin (PTX; 100 μM), field excitatory postsynaptic potentials (fEPSPs) were evoked by constant current stimulation (0.1 ms duration) of the Schaffer collaterals of CA3 pyramidal cells, *via* a bipolar platinum stimulating electrode placed into CA1 stratum radiatum. Whole-cell recordings of individual CA1 pyramidal cells were performed with patch pipettes filled with (in mM) 135 K-gluconate, 5 HEPES, 3 MgCl_2_, 5 EGTA, 2 Na_2_ATP, 0.3 Na_3_GTP, and 4 NaCl (pH 7.3) to monitor action potentials (APs) of CA1 pyramidal cells or excitatory synaptic events. To test cell excitability in current-clamp mode, a depolarizing ramp test was used to elicit action potentials from the initial membrane potential of −70 mV. Spontaneously occurring excitatory postsynaptic currents (spEPSCs) were monitored in voltage-clamp mode, with cells clamped at −70 mV and in the presence of PTX. In some experiments, miniature EPSCs (mEPSCs) were recorded with tetrodotoxin (TTX; 0.5 μM) in the perfusion solution to block action potential-dependent transmitter release. To promote the occurrence of EPSCs, KCl was elevated to 6 mM, together with modest changes in CaCl_2_ (3 mM) and MgCl_2_ (1 mM). To investigate inhibitory postsynaptic currents (IPSCs) in CA1 pyramidal cells, patch pipettes were filled with a CsCl-based solution (130 mM CsCl, 5 mM HEPES, 3 mM MgCl_2_, 5 mM EGTA, 2 mM Na_2_ATP, 0.3 mM Na_3_GTP, and 4 mM NaCl (pH 7.3). Spontaneous IPSCs (spIPSCs) and miniature IPSCs (mIPSCs) were recorded in the presence of the AMPA/NMDA glutamate receptor antagonist kynurenic acid (KA; 2 mM) in the bath, without and with TTX, respectively. Note that IPSCs were downward at −70 mV due to high Cl^–^ in the pipette. For all whole-cell configurations, membrane potentials were corrected for liquid junction potentials. Pipette resistance ranged between 3 and 5 MΩ with internal solution. Series resistance for voltage-clamp recordings were compensated by 60–80%.

Signals were filtered at 6 kHz (for action potentials) or 2 kHz (for synaptic events) and sampled at 20 kHz using a Multiclamp 700B amplifier together with Digidata 1,550 or 1,440 interface and pClamp10.6 software (Molecular Devices, CA, United States). A MiniDigi 1B or 1A and AxoScope 10.6 were used for low-resolution scope recording, sampled at 1 kHz. Data analysis was performed off-line with Clampfit 10.6. In addition to the number of APs during depolarizing ramp, the rheobase, that is the minimal current necessary to elicit 1st AP during ramps, was determined as current threshold. Spontaneous synaptic events were detected using an automated event detection algorithm. At least five recordings (20 s each) collected at various time points were used to quantify the frequency, amplitude and the kinetics of synaptic events. Rise time was measured from 10 to 90% of the peak response. Decay time constant tau, i.e., the time required to decay to 37% of peak amplitude, was obtained by fitting the averaged current with a single exponential function.

Drugs and chemicals were obtained from Tocris Bioscience (Bio-techne GmbH, Wiesbaden-Nordenstadt, Germany) and Sigma-Aldrich Chemie GmbH (Steinheim, Germany). The ASM inhibitor ARC39 was kindly provided by Dr. Christoph Arenz (Institute of Chemistry, Humboldt University, Berlin, Germany). Stock solution of ARC39 was prepared in distilled water at 1 mM, after overnight sonicating. ARC39 is a light-sensitive chemical compound, which was therefore stored in light-safe Eppendorfs.

Data are expressed as means ± SEM. OriginPro 2015G (OriginLab Corporation, Northampton, MA, United States) was used for statistics and figures. Statistical comparisons of data were performed using ANOVA or Student’s *t* test as appropriate. Significance was assumed for *p* < 0.05.

## Results

### ARC39 Augments Field Excitatory Postsynaptic Potentials at the Schaffer-CA1 Synapse

**FIGURE 1:**
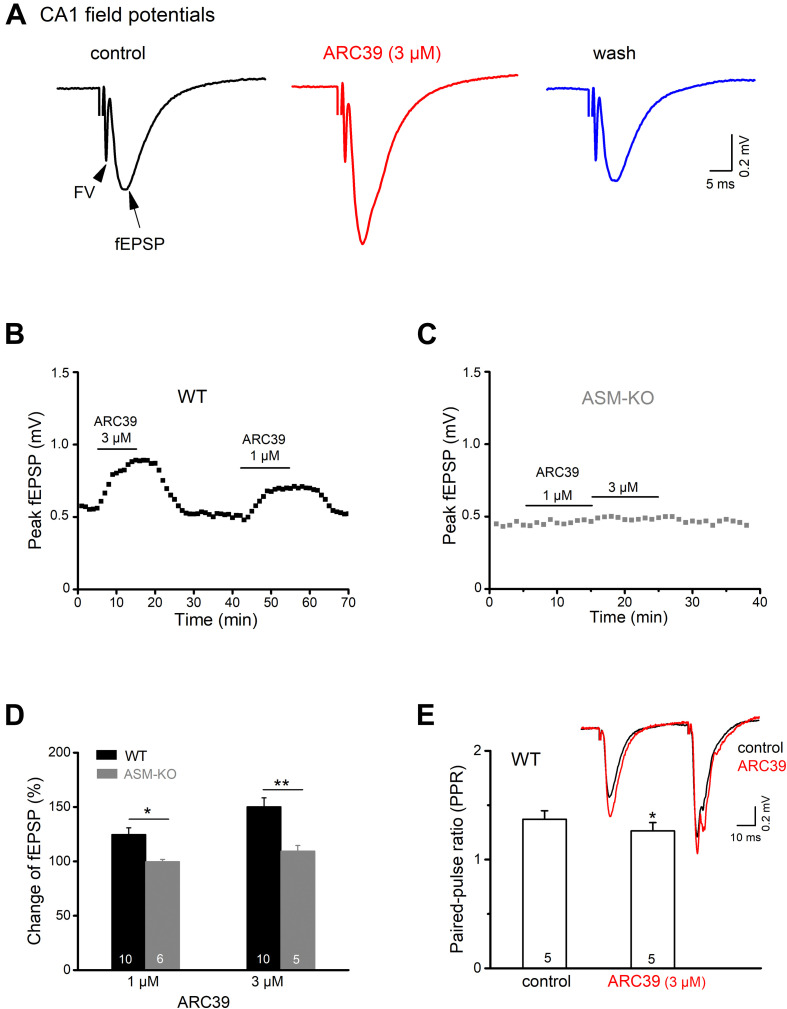
ASM inhibitor ARC39 enhances excitatory transmission at the Schaffer-CA1 synapse. Field potentials were recorded from CA1 stratum radiatum in the presence of picrotoxin (100 μM) to block GABA_A_ receptor-mediated inhibition. **(A)** Representative voltage traces from wild type (wt) slice (stimulation intensity 50 μA) before, during and after ARC39 (3 μM) application. Stimulation artifact was truncated. Field potentials in response to electrical stimulation of Schaffer collaterals consisted of axonal firing (fiber volley, FV) and subsequent synaptic response (field EPSP). **(B,C)** Time courses of ARC39-induced changes in peak amplitude of CA1 fEPSPs in a wt slice [from recording shown in panel **(A)**] and an ASM-KO slice. **(D)** Summary of dose-dependent effects of ARC39 on fEPSPs in wt and ASM-KO mice. **(E)** ARC39 reduced the paired-pulse ratio (PPR) of wt ventral CA1 fEPSP. Superimposed traces illustrate ARC39-induced effects on paired-pulse stimulation (40 μA, separated by 50 ms). PPR was quantified by dividing peak amplitude of 2nd fEPSP by that of 1st one. Numbers in columns indicate sample size. *p < 0.05, **p < 0.01.

To investigate how ASM affects excitatory neurotransmission between the CA3 and CA1 region of mouse ventral hippocampus, we performed electrical stimulation of the Schaffer collateral/commissural pathway in the presence of the GABA_A_ receptor antagonist PTX (100 μM) and monitored fEPSPs by means of an extracellular recording electrode located in CA1 stratum radiatum (Figure 1A). As illustrated in Figures 1A,B, bath application of the specific ASM inhibitor ARC39 (IC_50_ 0.02 μM for ASM *vs*. 100 μM for neutral SM; Roth et al., 2009; Arenz, 2010) at a concentration of 3 μM produced a rapid and reversible enhancement of the peak amplitude of fEPSPs from 0.54 ± 0.04 to 0.80 ± 0.06 mV (*n* = 10; paired *t*-test, *p* = 0.0001), corresponding to an increase to 150.33 ± 8.15 % of control (Figure 1D). To rule out that the rapid augmentation of fEPSPs was due to drug effects other than inhibition of ASM, we repeated the experiment in brain slices prepared from ASM knockout mice (ASM-KO). Reassuringly, ARC39 (3 μM) had no effect on CA1 fEPSPs in ventral slices from mutant mice (*n* = 5; from 0.47 ± 0.02 to 0.51 ± 0.02 mV; paired *t*-test, *p* = 0.144; Figures 1C,D), underscoring the specificity of the ASM inhibitor. Demonstrating the dose dependence of ARC39 action, application of a lower concentration (1 μM) produced a smaller, but still significant increase of fEPSP amplitude to 124.71 ± 6.32 % of control values (0.53 ± 0.04 mV, *n* = 10) in wt slices, but not in ASM-KO slices (*n* = 6; 99.72 ± 2.10 % of control value, which was 0.46 ± 0.02 mV) (Figures 1B–D).

To differentiate between a presynaptic vs. a postsynaptic site of drug action, we used a paired-pulse paradigm, where two identical electrical stimuli are delivered at a fixed time interval (here 50 ms). fEPSPs in area CA1 typically exhibit paired-pulse facilitation (PPF), meaning that the 2nd fEPSP is larger than the 1st one (inset in Figure 1E), yielding a paired pulse ratio (PPR) > 1. When determined in the presence of ARC39 (3 μM), we obtained a small, but significant decrease of PPR (peak amplitude of 2nd fEPSP divided by that of 1st fEPSP) from control 1.37 ± 0.08 to 1.26 ± 0.08 (*n* = 5, *p* = 0.047, paired *t*-test; Figure 1E). The fact that ARC 39 altered PPR points to a likely contribution of presynaptically located ASM to the regulation of synaptic strength at the Schaffer-CA1 synapse.

### ARC39 Enhances Spontaneous Excitatory Synaptic Events in Ventral CA1 Pyramidal Cells

**FIGURE 2:**
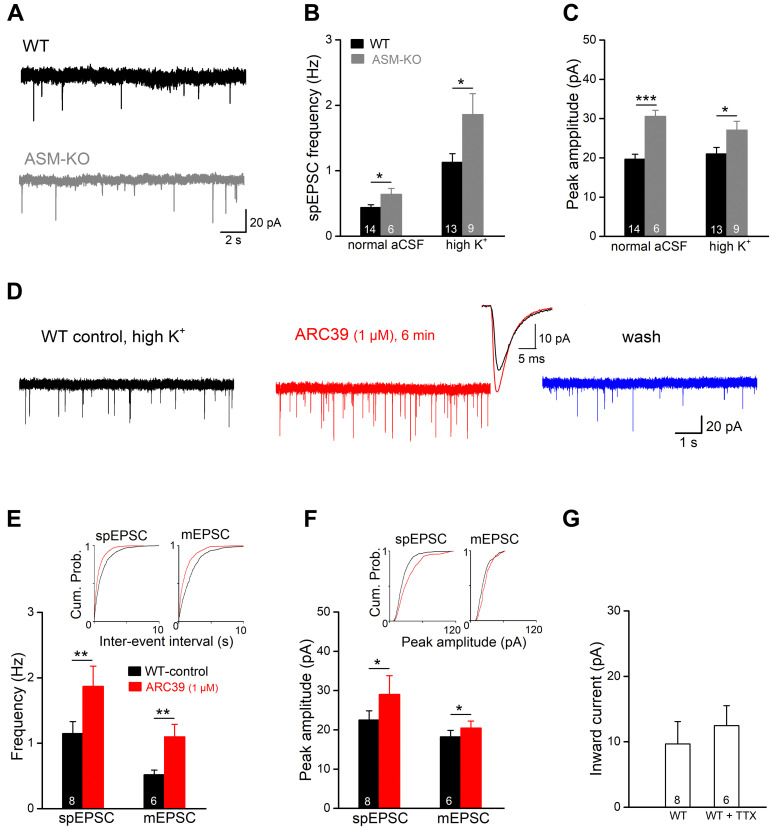
ARC39 increases frequency and amplitude of EPSCs in CA1 pyramidal cells. Spontaneously occurring excitatory postsynaptic events were recorded in the whole-cell voltage-clamp configuration at –70 mV in PTX (100 μM). Spontaneous EPSCs (spEPSC) and miniature EPSCs (mEPSCs) were collected in the absence and presence of TTX (0.5 μM), respectively. **(A)** Raw traces of spEPSCs from a wt cell (black trace) and an ASM-KO cell (gray trace) were collected in aCSF with normal K^+^ concentration (3 mM). **(B,C)** spIPSCs in ASM-KO pyramidal cells were more frequent and larger in amplitude in both solutions with normal K^+^ (3 mM) and with elevated K^+^ concentration (6 mM, to enhance synaptic events). **(D)** Raw traces of spEPSCs from a wt cell were collected in solution with high K^+^ (6 mM) before ARC39 application (control), 6 min in ARC39 (1 μM) and after 10 min of wash. Superimposed traces represent averaged events from same cell before (black trace) and during drug application (red trace). **(E–G)** Histograms summarize effects of acutely applied ARC39 (1 μM) on frequency **(E)** and averaged amplitude **(F)** of spEPSCs and mEPSCs in wt pyramidal cells, and concomitant shift of holding current **(G)**. Insets above in panels **(E,F)** are the cumulative probability plots for inter-event interval (reciprocal of frequency) and peak amplitude. *p < 0.05, **p < 0.01, and ***p < 0.001.

To investigate the effects of ASM inhibition on excitatory synaptic transmission at the cellular level, we recorded excitatory postsynaptic currents (EPSCs) in the whole-cell voltage-clamp configuration from ventral CA1 pyramidal cells. Holding potential (V_*h*_) was set to −70 mV and PTX (100 μM) was present to suppress GABA_A_ receptor-mediated currents. In the first set of experiments, we compared the frequency and amplitude of spontaneous EPSCs (spEPSCs) between wt and ASM-KO neurons (Figures 2A–C). In view of the rare occurrence of spEPSCs in wt cells with aCSF containing physiological [K^+^]_e_ of 3 mM (mean frequency 0.44 ± 0.04 Hz, *n* = 14), we increased [K^+^]_e_ to 6 mM (high K^+^ solution), which significantly increased spEPSC frequency (1.14 ± 0.13 Hz, *n* = 13; *p* = 0.0002). Interestingly, in both normal and high K^+^ solutions, ASM-KO neurons displayed altered spEPSC properties. Compared to their wt counterparts, ASM-KO spEPSCs occurred more frequently (normal aCSF: *n* = 6, 0.64 ± 0.09 Hz, *p* = 0.025; high K^+^: *n* = 9, 1.86 ± 32 Hz, *p* = 0.025; Figure 2B), and had a larger peak amplitude (normal aCSF: wt, 19.66 ± 1.25 pA, ASM-KO, 30.56 ± 1.55 pA, *p* = 0.0001; high K^+^: wt, 21.04 ± 1.63 pA, ASM-KO, 27.09 ± 2.22 pA, *p* = 0.036; Figure 2C). Elevation of [K^+^]_e_ accelerated the time course of spEPSC in wt neurons by shortening both rise time (from 1.32 ± 0.07 ms in normal aCSF to 0.98 ± 0.06 ms in high K^+^, *p* = 0.001) and decay time constant (from 4.86 ± 0.18 ms to 3.86 ± 0.20 ms, *p* = 0.001). In contrast to the significant effects on their frequency and amplitude, ablation of ASM did not affect the kinetics of spEPSCs in both normal aCSF and high K^+^ solution when compared to their wt counterparts (ASM-KO normal aCSF: rise time, 1.28 ± 0.18 ms, *p* = 0.808; decay tau, 5.09 ± 0.46 ms, *p* = 0.570; ASM-KO high K^+^: rise time, 1.09 ± 0.08 ms, *p* = 0.265; decay tau, 4.27 ± 0.29 ms, *p* = 0.247).

Next, we tested the effect of acute ASM suppression on spEPSCs of ventral CA1 pyramidal cells in wt slices in high K^+^ aCSF (Figure 2D). Since ARC39 at 1 μM produced robust effects on CA1 fEPSPs (Figure 1), the following experiments were performed with this concentration. Perfusion of ARC39 (1 μM) rapidly increased the frequency of spEPSCs from 1.15 ± 0.18 Hz in control to 1.87 ± 0.31 Hz (*n* = 8, *p* = 0.003, paired *t*-test; 162.45 ± 11.79 % of control value), together with increase in the averaged peak amplitude from 22.52 ± 2.31 pA to 29.02 ± 4.77 pA (*p* = 0.040, paired *t*-test; 124.22 ± 7.36 % of control value) (Figures 2D–F). Calculation of spEPSC kinetics revealed no significant change in response to ARC39 (10–90% rise time from 0.98 ± 0.07 ms to 0.90 ± 0.07 ms, *p* = 0.346; decay time constant from 3.66 ± 0.26 ms to 3.59 ± 0.24 ms, *p* = 0.694). Again, the effect of ARC39 on spEPSCs could be causally linked to inhibition of ASM, because the drug failed to affect spEPSCs in ASM-KO pyramidal cells (*n* = 6, control, 0.64 ± 0.09 Hz; ARC39, 0.58 ± 0.09 Hz, *p* = 0.119, normal aCSF). When TTX (0.5 μM) was used to capture only synaptic release events independent from presynaptic action potentials, so-called miniature EPSC (mEPSC), ARC39 (1 μM) strongly enhanced their frequency (from 0.52 ± 0.07 Hz to 1.10 ± 0.19 Hz, *n* = 6, *p* = 0.009; 219.42 ± 37.19 % of control value), whereas the increase in peak amplitude was less pronounced (from 18.24 ± 1.62 pA to 20.48 ± 1.75 pA, *p* = 0.016; 112.68 ± 3.46 % of control value) (Figures 2E,F). Notably, the ARC39-induced changes in EPSC properties were accompanied by an appreciable inward shift of holding current independent of the absence or presence of TTX (Figure 2G).

### ARC39 Promotes Inhibitory Synaptic Inputs Onto Ventral CA1 Pyramidal Cells

**FIGURE 3:**
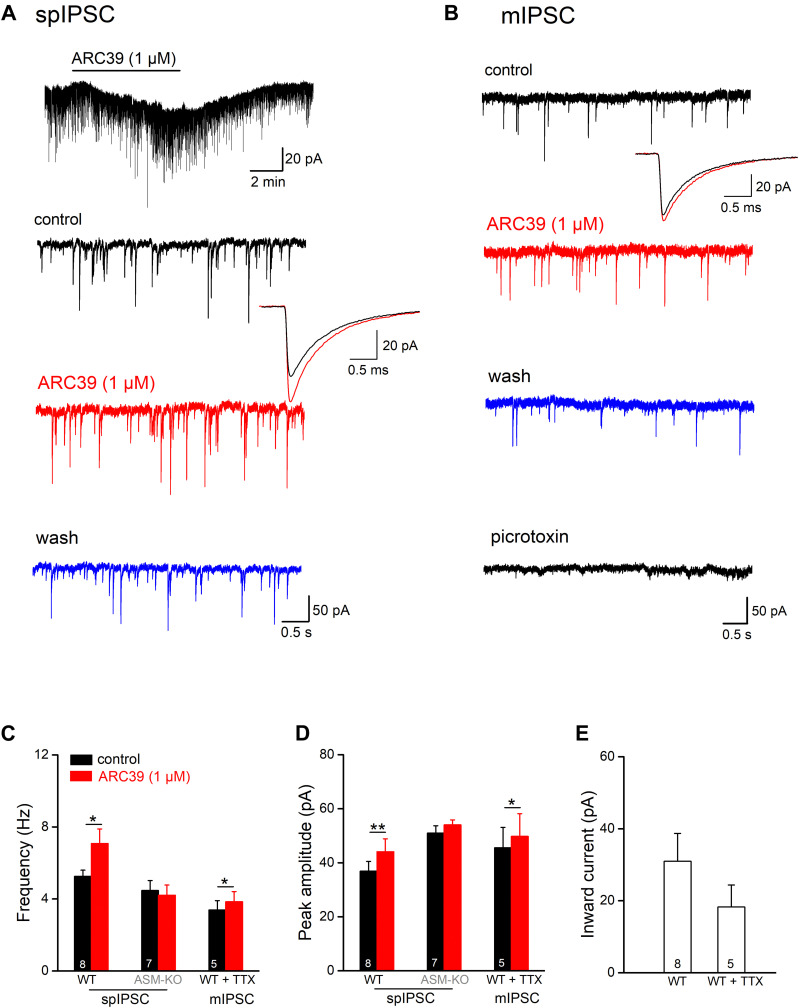
ARC39 augments frequency and amplitude of IPSCs in CA1 pyramidal cells. Spontaneously occurring inhibitory synaptic events were recorded with CsCl-filled pipettes in the presence of KA (2 mM) to block ionotropic glutamate receptors at –70 mV. **(A,B)** Raw current traces illustrate the facilitating effects of ARC39 on spontaneous IPSCs [spIPSCs; **(A)**] and miniature IPSCs [mIPSCs, in 0.5 μM TTX; **(B)**] in two pyramidal cells from wt slices. The trace above in panel **(A)** is a scope recording showing the time course of holding current. spIPSCs and mIPSCs were collected before ARC39 application (control), 5–6 min in ARC39 (1 μM, red trace), and after 8–10 min of washout (blue trace). The averaged events before (black trace) and during ARC39 application (red trace) were enlarged and superimposed to depict the kinetics of IPSCs. At the end of the experiment in panel **(B)**, PTX (100 μM) was applied to verify the GABAergic nature of spontaneous events. **(C,D)** Histograms summarize effects of acute ARC39 (1 μM) on frequency **(C)** and averaged amplitude **(D)** of spIPSCs and mIPSCs. **(E)** Quantification of ARC39-induced change in holding current without and with TTX (0.5 μM). Note broad suppression of K^+^ channels by high Cs^+^-based pipette solution. *p < 0.05, and **p < 0.01.

In addition to excitatory synaptic projections, hippocampal pyramidal cells receive strong inhibitory inputs involving GABAergic interneurons in both feed-forward and feed-back circuits. To examine whether ASM might also modulate GABAergic inputs, we monitored spontaneous inhibitory postsynaptic currents (spIPSCs) in ventral CA1 pyramidal cells voltage-clamped at −70 mV. spIPSCs were pharmacologically isolated by adding the ionotropic glutamate receptor blocker kynurenic acid (KA, 2 mM) to the bathing solution. Due to the high Cl^–^ concentration in the recording pipette solution, spIPSCs were inward (Figure 3A). Perfusion of control slices with ARC39 (1 μM) produced a rapid and reversible inward shift of holding current amounting to 30.96 ± 7.76 pA (*n* = 8; Figures 3A,E), which would correspond to an increase in tonic GABA inhibition under physiological conditions. Concomitantly, we observed an increase in the frequency of spIPSC from 5.26 ± 0.34 to 7.08 ± 0.81 Hz (*n* = 8; *p* = 0.011, paired *t*-test) and in their peak amplitude from 36.87 ± 3.65 pA to 44.10 ± 4.76 pA (*n* = 8; *p* = 0.009, paired *t*-test, Figures 3A,C,D). Expressed as percentage change from control, ARC39 enhanced spIPSC frequency by 32.52 ± 7.06 % and amplitude by 18.90 ± 4.10%. As seen for the time course of EPSCs before, ARC39 did not alter IPSC kinetics including 10–90% rise time (control, 0.58 ± 0.02 ms; ARC39, 0.56 ± 0.03 ms; *p* = 0.433) and decay time constant (control, 7.93 ± 0.25 ms; ARC39, 7.83 ± 0.32 ms; *p* = 0.653). When we isolated the action potential-independent fraction of GABA release events in control pyramidal cells with TTX to obtain miniature IPSCs (mIPSCs), the augmenting effect of acute ASM suppression was still detectable, albeit smaller in size, yielding a slight but significant increase in mIPSC frequency (*n* = 5; from 3.39 ± 0.52 Hz to 3.85 ± 0.56 Hz, *p* = 0.010) and peak amplitude (from 45.56 ± 7.55 pA to 49.80 ± 8.36 pA, *p* = 0.037) (Figures 3B–D). The effects on mIPSCs were accompanied again by an inward shift of holding current (18.28 ± 6.11 pA; Figure 3E). In some experiments, PTX (100 μM) was applied at the end of the experiment to demonstrate the GABAergic nature of mIPSCs (Figure 3B). Notably, the ARC39-induced increase in mIPSC frequency (13.36 ± 2.14 %) was more moderate than in spIPSCs (32.52 ± 7.06 %; *p* = 0.042), suggesting that ARC39 promotes firing of GABAergic neurons.

In hippocampal slices from ASM-KO mice, ARC39 (1 μM) failed to change spIPSC frequency and amplitude (*n* = 7; Figures 3C,D). Note that, while the frequency of spIPSCs in ASM-KO pyramidal cells (4.47 ± 0.55 Hz, *n* = 7) was comparable with that in wt neurons (5.26 ± 0.34 Hz; *p* = 0.231), the amplitude of ASM-KO spIPSCs was significantly larger than that in wt neurons (ASM-KO, 50.97 ± 2.73 pA; wt 36.87 ± 3.65 pA; *p* = 0.005). Compared to wt spIPSCs, spIPSCs in ASM-KO cells had similar rise time (0.50 ± 0.08 ms; *p* = 0.066 *vs* wt-spIPSC) and decay time constants (7.73 ± 0.49 ms, *p* = 0.715 *vs* wt-spIPSCs), and were not altered by ARC39 (*data not shown*).

### ARC39 Reduces Ventral CA1 Pyramidal Cell Excitability

**FIGURE 4:**
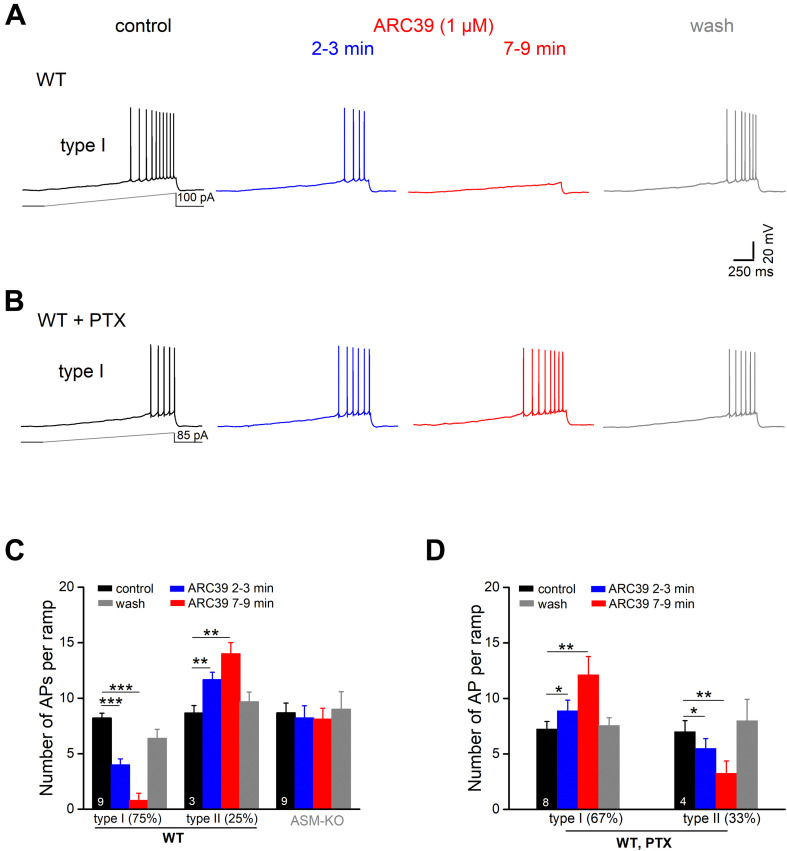
Predominant suppression of CA1 pyramidal cell firing by ARC39 involves GABAergic inhibition. Action potentials (APs) of ventral CA1 pyramidal cells were evoked in current-clamp mode by depolarizing voltage ramp rising from 0 to 50–100 pA within 2 s. All cells were initially held at –70 mV, before drug application. **(A)** Representative voltage traces taken before, during and after ARC39 (1 μM for 10 min) illustrate gradual and reversible reduction of AP firing in a wt cell. **(B)** Representative traces showing how inhibition of GABA_A_ receptors by PTX (100 μM) reversed effect of ARC39 from strong suppression to moderate excitation. **(C)** Summary of effects of ARC39 on AP discharges in wt and ASM-KO pyramidal cells. **(D)** Reversed response pattern of wt cells to ARC39 in PTX. *p < 0.05, **p < 0.01, and ***p < 0.001.

To investigate the impact of ASM on pyramidal cell excitability, we performed whole-cell recordings in current-clamp mode. Ventral CA1 pyramidal cells from wt and ASM-KO mice had a comparable resting membrane potential (wt, *n* = 59, −71.99 ± 0.46 mV; ASM-KO, *n* = 41, −71.93 ± 0.42 mV, and *p* = 0.934). When membrane potential was held at −70 mV by current injection, wt and ASM-KO pyramidal cells had very similar input resistance (R_N_; wt, 205.61 ± 5.78 MΩ; ASM-KO, 214.66 ± 6.85 MΩ, and *p* = 0.416), and displayed a virtually identical pattern of action potential (AP) firing in response to depolarizing current ramps from 0 to 100 pA over 2 s (wt, 14.13 ± 0.92 APs per ramp; ASM-KO, 16.05 ± 1.39 APs per ramp, *p* = 0.234). Further characterization of the first AP during the ramp test revealed no detectable change in amplitude, threshold and half-width between the two groups of cells (Table 1). These data suggest that constitutive loss of the enzyme did not appear to engender lasting alterations of basic firing properties in young adult ASM-KO mice. In striking contrast, acute suppression of ASM activity by ARC39 strongly suppressed pyramidal cell firing, often to the extent that the depolarizing ramp failed to elicit even a single AP (Figures 4A,C). In this set of experiments, the depolarizing current injected during ramp protocol (from 0 to 50–120 pA) was adjusted individually to evoke 5–10 APs per ramp before drug application (control), starting always from a membrane potential pre-set to −70 mV. In the majority of cells (9 out of 12, i.e., 75.0%; type I in Figure 4C), ARC39 (1 μM) produced a rapid hyperpolarization, which reached its maximum effect after 7–9 min of bath application (from initial −70 to −74.33 ± 0.68 mV) and was accompanied by a significant reduction in R_N_ (control, 153.11 ± 10.17 MΩ; ARC39 132.44 ± 9.82 MΩ; *p* = 0.0002). Concurrently, the number of APs during ramp depolarization declined from 8.22 ± 0.43 to 0.78 ± 0.66 (*n* = 9, *p* = 0.0001) (Figures 4A,C). The strong dampening effect of ARC39 on pyramidal cell excitability was reversible upon drug-washout (Figures 4A,C). The remaining few pyramidal cells (3 out of 12, i.e., 25.0%; type II in Figure 4C) responded to ARC39 with an increase in AP firing and with membrane depolarization. Demonstrating again its specificity, ARC39 (1 μM) failed to change membrane potential (−70.24 ± 0.32 mV), AP discharges (control, 8.67 ± 0.88; ARC39, 8.11 ± 0.98; *p* = 0.139, Figure 4C) and R_N_ (control, 139.70 ± 7.92 MΩ; ARC39, 142.89 ± 7.92 MΩ; *p* = 0.461), when examined in ventral CA1 pyramidal cells (*n* = 9) from ASM-KO mice (Figure 4C).

**TABLE 1:** Properties of ventral hippocampal CA1 pyramidal cells in wild type and ASM-KO mice.

**Genotype**	**RMP (mV)**	**Cm (pF)**	RN **(MΩ)**	**# of APs per ramp (0–100 pA)**	**Rheobase (pA)**	**Voltage Threshold (mV)**	**AP Amplitude (mV)**	**AP ½ Width (ms)**
WT	−71.99	92.70	205.61	14.13	58.65	−51.90	97.71	0.91
(*n* = 59)	±0.46	±2.78	±5.78	±0.92	±1.98	±0.34	±0.51	±0.01
ASM-KO	−71.93	86.63	214.66	16.05	54.40	−51.84	97.85	0.94
(*n* = 41)	±0.42	±3.11	±6.85	±1.39	±2.46	±0.41	±0.57	±0.02

*Data are presented as mean ± SEM. Except for resting membrane potential (RMP), passive and active membrane properties were characterized from a membrane potential of −70 mV. The first action potential (AP) elicited by the depolarizing ramp current (0–100 pA in 2 s) was used for analysis.*

We next asked whether the predominantly dampening effect of ARC39 was achieved through the strong increase in GABAergic inhibition reported above (Figure 3), or through interference of the drug with the neurons’ intrinsic electrophysiological properties, or through both. To address the former possibility, we repeated the ramp protocol in the presence of PTX (100 μM). With GABA_A_ receptors blocked, the cessation of firing in response to ARC39 was completely abrogated in 8 out of 12 neurons, i.e., 66.7% (Figure 4B, type I in Figure 4D), and the majority of cells even discharged APs at a higher rate. The ARC39-induced increase in AP number per ramp in type I cells (*n* = 8, from 7.25 ± 0.67 to 12.13 ± 1.64, *p* = 0.005) was accompanied by membrane depolarization of 2.39 ± 0.80 mV (7–9 min of drug application). In the few neurons exhibiting an attenuated firing response to ARC39 in PTX (type II in Figure 4D, *n* = 4 out of 12, i.e., 33.3%; from 7.00 ± 1.00 APs/ramp to 3.25 ± 1.11 APs/ramp, *p* = 0.009), membrane potential was concomitantly hyperpolarized by 1.45 ± 0.41 mV.

**FIGURE 5:**
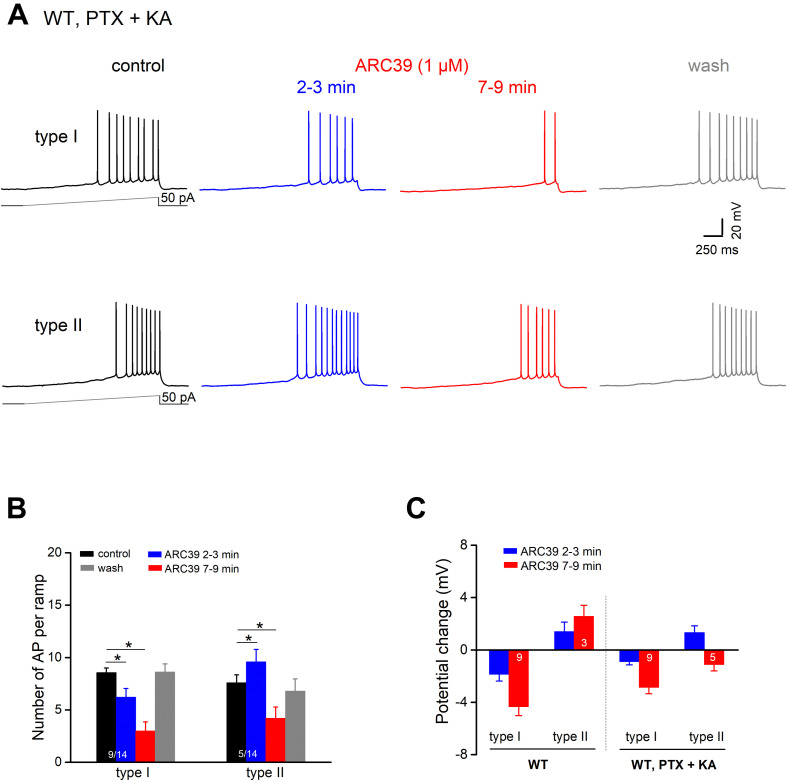
ARC39 attenuates intrinsic firing properties of CA1 pyramidal cells. All current-clamp recordings were performed in wt cells, which were pharmacologically isolated from GABAergic and glutamatergic synaptic inputs by PTX (100 μM) and KA (2 mM), respectively. **(A)** Recordings from two CA1 pyramidal cells showing the two types of responses to ARC39 (1 μM). Type I was characterized by gradual decline in firing during drug application, whereas type II displayed an early transient increase in firing before the reduction set in. **(B,C)** Histograms summarize the divergent effect of ARC39 on AP firing **(B)** and concurrent change in membrane potential **(C)**, sub-grouped according to their earlier response. The corresponding recordings from wt cells obtained in the absence of PTX and KA were included in panel **(C)** (left-hand side) for comparison. *p < 0.05.

To determine whether ARC39 has also a direct impact on the intrinsic excitability of pyramidal cells, we functionally isolated the neurons from their main excitatory and inhibitory synaptic inputs by adding the ionotropic glutamate receptor agonist kynurenic acid (KA) and PTX, respectively, to the bathing solution. Under these conditions, we observed two different responses of ventral CA1 pyramidal cells to ARC39 (1 μM, Figures 5A,B). Whereas the majority of cells exhibited a gradual decline in firing in the course of drug application (*n* = 9 out of 14, 64.3 %; type I), a minority showed an initial increase within 2–3 min of ARC39, followed by a decrease (*n* = 5, 35.7 %; type II). The ARC39-induced changes in APs were accompanied by corresponding shifts in membrane potentials, i.e., depolarization coincided with increased firing and hyperpolarization with reduced firing (Figures 5B,C). However, regardless of the initial divergence, the final effect of ARC39 on AP firing after 7–9 min of bath application was uniformly inhibitory in nature, with AP discharges reduced from 8.29 ± 0.40 per ramp to 3.43 ± 0.66 (*n* = 14, *p* = 0.0001) and R_N_ reduced from 178.00 ± 10.90 MΩ to 157.40 ± 12.00 MΩ (*p* = 0.005).

When comparing the mitigating action of ARC39 on the firing propensity of pyramidal cells between those having intact synapses and those with pharmacologically blocked synapses, the drug seemed to be more efficient in the former. For example, the drug completely abrogated ramp-evoked firing in 7 out of 9 CA1 pyramidal cells receiving normal synaptic input (Figure 4A), whereas residual AP firing was maintained in 13 out of the 14 cells in input-deprived neurons (Figure 5A). Furthermore, the ARC39-evoked membrane hyperpolarization in the latter cells amounted to only 2.24 ± 0.41 mV (*n* = 14, 7–9 min), which was significantly smaller than that in cells embedded in an operative network (4.33 ± 0.68 mV; *p* = 0.029) (Figure 5C).

## Discussion

Aberrant activity of the ASM/ceramide system has been recognized as a major factor in the pathogenesis of depression, but little is known about how ASM regulates neuronal activity in the hippocampus, arguably the most intensively studied brain region in the context of neural plasticity, neurogenesis and affective disorders. Within the hippocampus, its ventral part has taken center stage in depression and anxiety research, as it is, more than the dorsal part, prominently involved in the processing of emotional content and the control of affective behavior (see Introduction). The ventral hippocampus is also particularly sensitive to sphingolipid modulation by depression-associated rises in corticosterone levels, causing alterations in the regional content and distribution of ceramide, dihydrosphingomyelin and phosphatidic acid (Miranda et al., 2019).

We therefore chose to investigate the effects of ASM on neuronal excitability in mouse brain slices containing the ventral hippocampus. We pursued distinct strategies to assess the impact of ASM at short notice as well as in the long run. For brief suppression of ASM activity, we applied the highly selective small molecule inhibitor ARC39 (IC_50_ 0.02 μM for ASM *vs*. 100 μM for neutral SM; Roth et al., 2009; Arenz, 2010), for constitutive ablation of ASM, we used hippocampi from ASM-KO mice and compared them to their wt counterparts. It should be noted that ASM-KO mice lack both L-ASM and S-ASM, whereas brief bath application (≤10 min) of the ASM inhibitor ARC39 should confine the drug effect to suppression of S-ASM. Being a bisphosphonate, ARC39 is highly negatively charged at physiological pH and thus unlikely to enter cells by passive diffusion (Naser et al., 2020). Thus, it seemed reasonable to expect much more pronounced effects in the ASM-deficient hippocampus as compared to the short pharmacological inhibition of S-ASM alone.

To our surprise, it turned out to be the other way around, especially when we probed pyramidal cell firing. Whereas ventral CA1 pyramidal cells from wt and ASM-KO were virtually indistinguishable with respect to resting membrane potential, input resistance and depolarization-induced firing, ARC39 rapidly and strongly dampened cell excitability in most neurons. Concerns that this highly unexpected action of ARC39 might represent a side effect not associated with ASM inhibition were dispelled by the complete lack of drug effect in neurons from ASM-KO mice. The question remains, though, why disruption of the ASM gene did not leave a signature on firing properties, whereas a brief inhibition restricted to S-ASM almost entirely wiped out action potential discharges. Since finely tunable firing is essential to the proper functioning of the nervous system, it seems plausible to assume that, during development, the intrinsic electrophysiological properties of ASM-deficient neurons had been re-adjusted to normal level in a homeostatic fashion.

In view of the rapid and reversible action of ARC39, it is tempting to speculate that (i) the extracellular space of hippocampal tissue should contain a sufficient concentration of S-ASM (of unknown source), that (ii) S-ASM should be tonically active, and that (iii) this tonic activity enables the enzyme to control the level of cellular excitability. If we further entertain this hypothesis, a number of questions come to mind. First and foremost, how can S-ASM be enzymatically active in the extracellular space, given that its pH optimum is around 4.5–5.0 (Kornhuber et al., 2015). It has been reported that S-ASM can hydrolyze physiologic sphingomyelin-containing substrates at neutral pH, provided that the orientation of sphingomyelin in the substrate allows a ready interaction with the enzyme (Schissel et al., 1998), but whether this notion holds in our preparation remains to be established. Alternatively, S-ASM may become activated in acidic microdomains that are found along distal processes of oligodendrocytes (Ro and Carson, 2004). Second, what is the source and role of Zn^2+^, which is required for S-ASM activity (Schissel et al., 1998)? Zn^2+^ accumulates in glutamate-containing synaptic vesicles of excitatory neurons of the hippocampus and other telencephalic structures (Smart et al., 2004). Depending on the strength and duration of synaptic stimulation, estimates of free Zn^2+^ concentration in the synaptic cleft range between 10–100 μM (Smart et al., 2004). Importantly, Zn^2+^ may spread up to 100 μm from its release site, attaining concentrations of up to 5–30 μM at distant sites (Ueno et al., 2002). Such a scenario opens the intriguing possibility that S-ASM—and, thus, the excitability of the postsynaptic cell—may be regulated in an activity-dependent fashion through the extent of Zn^2+^ released from presynaptic vesicles. The third question relates to the mechanisms through which S-ASM modulates cell firing. It is worth remembering that, with its catalytic site facing the outer leaflet of the membrane, S-ASM converts sphingomyelin into ceramide in the outer layer. Due to its bulky head group, the ceramide should stay in the outer leaflet, where it is produced (Kornhuber et al., 2015). With more ceramide accumulating in its extracellular leaflet, the cell membrane exhibits a negative curvature, which facilitates endocytosis, possibly removing ion channels, receptors, transporters and pumps from cell surface (Trajkovic et al., 2008). Furthermore, changes in ceramide content would be expected to influence the formation of ceramide-rich signaling platforms, which should have a direct or indirect impact on the membrane-bound molecules that govern the neuron’s firing behavior.

While we cannot pinpoint the molecular targets affected by S-ASM in the native hippocampus, a number of ion channels have been already shown as being susceptible to ceramide, including K^+^ channels (Kir, Kv1.3, Kv2.1, and HERG), and Cl^–^ channels (CFTR) (Bock et al., 2003; Rosenhouse-Dantsker et al., 2012). Sphingosine-1-phosphate (S1P), a downstream product of ceramide breakdown, binds to G protein-coupled receptors (S1P1, S1P2, S1P3, and S1P5) to influence various channels, such as cation and Ca^2+^ channels (TRPC1/4/5, TRPM3, and VDCC), K^+^ channels (Kir and BK) and Cl^–^ channels (Rosenhouse-Dantsker et al., 2012). Previous work has demonstrated that ablation of the S1P3 receptor impairs the excitability of hippocampal CA3 pyramidal cells (Weth-Malsch et al., 2016). *Vice versa*, when sphinogomyelin hydrolysis was driven by treating hippocampal slices with neutral sphingomyelinase (NSM), Norman et al. (2010) observed an increased excitability of CA1 pyramidal cells through a S1P-mediated mechanism. Finally, a recent study demonstrated that ASM modulates the biophysical properties of TRPC6 activity by controlling the interaction of TRPC6 channels with specific lipid subdomains (Zeitler et al., 2019). The apparent multitude of ion channels, whose gating characteristics as well as surface expression may be affected by S-ASM, may account for the different types of altered firing patterns in response to acute ASM inhibition that we observed in ARC39-treated neurons (Figures 4, 5). It seems therefore conceivable that, depending on which set of ion channels is expressed and active in the plasma membrane of an individual neuron at a given time, acute suppression of ASM activity and the concomitant changes in the levels of lipids upstream and downstream of the enzyme might produce different actions on neuronal firing.

In addition to direct effects of ARC39 on the intrinsic excitability of the postsynaptic neuron, suppression of S-ASM entailed also consequences for both inhibitory and excitatory synapses. ARC39 produced a significant increase in the frequency and amplitude of spontaneous and miniature IPSCs, thereby making a major contribution to the control of pyramidal cell excitability. At excitatory synapses, S-ASM had a strong attenuating effect, which could be released by ARC39 in a dose-dependent and reversible fashion, as demonstrated in fEPSP recordings from CA1 stratum radiatum and in spontaneous and miniature EPSCs recordings from voltage-clamped CA1 pyramidal cells. Again demonstrating its specificity, ARC39 failed to affect any of the features of glutamatergic and GABAergic synapses in ASM-deficient hippocampi. It is worth noting, however, that, when compared to wt controls, spEPSCs from ASM-KO neurons occurred at a significantly higher frequency and had a significantly larger amplitude, consistent with the effects of ARC39 in wt hippocampi. Regarding GABAergic inhibition, the difference between wt and ASM-KO was less conspicuous, concerning only amplitude, which was increased, as it was by ARC39, but not frequency of spIPSCs. These data suggest that, in apparent contrast to the intrinsic electrophysiological properties of CA1 pyramidal cells, the synaptic connectivity of ASM-deficient hippocampi had not been fully re-adjusted to normal physiological levels.

The fact that ARC39 increased the frequency of both mIPSCs and mEPSCs points to a presynaptic component of tonic S-ASM activity at inhibitory and excitatory transmission sites, respectively. This conclusion would be consistent with previous findings showing that changes in sphingolipid composition and signaling impacts transmitter release. For example, Kanno et al. (2010) reported that addition of S1P increased the rate of AMPA receptor-mediated mEPSCs. In synaptosomal preparations from ASM-KO hippocampi, Camoletto et al. (2009) observed a threefold increase in sphingomyelin levels and a four- to fivefold increase in sphingosine levels compared to wild type, whereas ceramide levels remained unaffected. As a functional consequence of the profoundly altered synaptic membrane lipid composition in ASM-KO hippocampi, they found that Munc18- and syntaxin1-mediated mechanisms of synaptic vesicle docking were impaired.

In conclusion, our data suggest that the tonic activity of S-ASM appears to put a brake on synaptic excitation in pyramidal cell dendrites, whereas GABA_A_ receptor-mediated transmission is attenuated concomitant with an increase in (axo-)somatic excitability. Thus, S-ASM activity may be strategically well poised to regulate several defining features of hippocampal network activity.

## Data Availability Statement

The raw data supporting the conclusions of this article will be made available by the authors, without undue reservation.

## Ethics Statement

The animal study was reviewed and approved by Local Government of Lower Franconia, Würzburg.

## Author Contributions

CA and JK initiated and supervised the project. FZ designed the experiments, performed data analysis, and prepared the figures. C-HL and FZ performed whole-cell recordings of action potential firing and EPSCs. FZ conducted field potential and IPSCs recordings. CA wrote the manuscript with contributions from FZ. All authors contributed to the article and approved the submitted version.

## Conflict of Interest

The authors declare that the research was conducted in the absence of any commercial or financial relationships that could be construed as a potential conflict of interest.

## Abbreviations

aCSF: artificial cerebrospinal fluid
AP: action potential
ASM: acid sphingomyelinase
EPSC: excitatory postsynaptic current
fEPSP: field excitatory postsynaptic potential
IPSC: inhibitory postsynaptic current.
